# Developing an Asymmetry Method for Detecting Postictal Hyperperfusion in Poststroke Epilepsy

**DOI:** 10.3389/fneur.2022.877386

**Published:** 2022-07-13

**Authors:** Kazuki Fukuma, Tomotaka Tanaka, Shigetoshi Takaya, Maya Tojima, Katsuya Kobayashi, Akihiro Shimotake, Yoshiaki Morita, Kunihiro Nishimura, Masatoshi Koga, Kazunori Toyoda, Riki Matsumoto, Ryosuke Takahashi, Akio Ikeda, Masafumi Ihara

**Affiliations:** ^1^Department of Neurology, National Cerebral and Cardiovascular Center, Osaka, Japan; ^2^Department of Neurology, Senri Rehabilitation Hospital, Osaka, Japan; ^3^Department of Rehabilitation Medicine, Senri Rehabilitation Hospital, Osaka, Japan; ^4^Department of Neurology, Kyoto University Graduate School of Medicine, Kyoto, Japan; ^5^Department of Radiology, National Cerebral and Cardiovascular Center, Osaka, Japan; ^6^Departments of Preventive Medicine and Epidemiology, National Cerebral and Cardiovascular Center, Osaka, Japan; ^7^Department of Cerebrovascular Medicine, National Cerebral and Cardiovascular Center, Osaka, Japan; ^8^Division of Neurology, Kobe University Graduate School of Medicine, Kobe, Japan; ^9^Department of Epilepsy, Movement Disorders and Physiology, Kyoto University Graduate School of Medicine, Kyoto, Japan

**Keywords:** asymmetry, epilepsy, hyperperfusion, poststroke epilepsy, single photon emission computed tomography (SPECT), stroke

## Abstract

Using dual single-photon emission computed tomography (SPECT) scanning, we recently found the postictal-interictal (P-I) subtraction method frequently detects prolonged postictal hyperperfusion in poststroke epilepsy (PSE) and thus may be valuable for auxiliary diagnosis. This study aimed to determine if the asymmetry method can localize hyperperfusion to reflect epileptic activity in PSE using a single postictal SPECT scan. Sixty-four patients with PSE who had undergone perfusion SPECT two times (postictal and interictal) were enrolled. We formulated a novel asymmetry method (subtraction analysis of reversed postictal SPECT from postictal SPECT, co-registered to magnetic resonance imaging) to identify paradoxical asymmetric increase, defined as a higher perfusion area adjacent to stroke lesions compared to the contralateral side. The postictal hyperperfusion area and detection rates were determined by the asymmetry and P-I subtraction methods independently. We subsequently calculated the sensitivity and specificity of the asymmetry method, compared to the gold standard P-I subtraction method. We also evaluated lateralization concordance between the asymmetry method and other clinical findings. Among 64 patients (median age, 75 years), prolonged postictal hyperperfusion was detected in 43 (67%) by the asymmetry, and 54 (84%) the P-I, method. The asymmetry method had high sensitivity (80%) and specificity (100%) in detecting postictal hyperperfusion, showing high lateralization concordance with seizure semiology (97%) and epileptiform electroencephalography findings (interictal/ictal epileptiform discharges or periodic discharges) (100%). The present study demonstrated the advantages of the objective asymmetry method for detecting prolonged hyperperfusion through using one postictal SPECT scan in PSE.

## Introduction

Poststroke epilepsy (PSE) is the most common type of epilepsy in the elderly (1) and is related to poor prognosis in poststroke survivors (2). Diagnosis of PSE is often challenging in cases which present with non-convulsive seizures and show no epileptiform abnormalities on electroencephalography (EEG) (3). Our recent study identified prolonged postictal hyperperfusion in PSE using postictal-interictal (P-I) subtraction perfusion single-photon emission computed tomography (SPECT) co-registered to magnetic resonance imaging (MRI). The P-I subtraction method can detect even mild increases in perfusion between the postictal and interictal phase, achieving a high detection rate (86%) for postictal hyperperfusion, and high intra- and interobserver variability (Kappa statistic of 0.92 and 0.85, respectively), ensuring objectivity and reproducibility. Furthermore, our study demonstrated postictal hyperperfusion is prolonged for hours, even days, in PSE. The laterality of the hyperperfusion area on the P-I subtraction method, had a high concordance rate with the laterality of stroke lesions (98%), seizure symptoms (92%), and epileptiform EEG findings (100%), suggesting a trace after spreading epileptic activity and a possible diagnostic finding for PSE (4). However, the P-I subtraction method needs both postictal and interictal images. The impact of radiation exposure and cost also must be considered. Therefore, a practical method for the detection of hyperperfusion using only postictal perfusion images is warranted.

An asymmetry method, comparing ipsilateral and contralateral regions, can be performed by single acquisition of brain images as a clinically feasible method. Previous studies have followed this approach using an asymmetry index with a manual definition of the region of interest (5–9). This has been exploited for the detection of the epileptic focus with hypoperfusion or hypometabolism in interictal SPECT (5) or ^18^ F-fluorodeoxyglucose positron emission tomography (6, 7) and hyperperfusion in internal carotid artery stenosis with post-surgery SPECT (8, 9). However, its applicability in the diagnosis of epilepsy in comparison to P-I subtraction methods has not been established.

Asymmetry of cerebral perfusion in poststroke patients without epilepsy should arise from lower perfusion in the hemisphere ipsilateral to the stroke lesions, particularly in adjacent, rather than contralateral areas (10, 11). However, our previous study in PSE showed prolonged postictal hyperperfusion occurred locally within hypoperfused areas adjacent to the stroke lesions, akin to “islands in the sea” (4). The asymmetry method may thus be the optimal approach for detecting higher perfusion in the areas adjacent to stroke lesions, rather than the contralateral side (i.e., paradoxical asymmetric increase) in PSE. We have thus developed the asymmetry method, incorporating an objective technique, for the analysis of postictal SPECT images in PSE.

This study aimed to determine if the asymmetry method using a single postictal SPECT scan is more effective than the subtraction method in the localization of hyperperfusion as a marker of epileptic activity in PSE.

## Methods

### Study Protocol

This study was conducted as a single-center sub-analysis of the PROgnosis of Post-Stroke Epilepsy (PROPOSE) study (UMIN000019940), consisting of (i) a preceding single-center retrospective cohort in the National Cerebral and Cardiovascular Center (NCVC) between January 2011 and October 2014 and (ii) a multi-center (eight sites) prospective cohort between November 2014 and September 2018 (3). PROPOSE was a non-interventional observational cohort study, enrolling patients with PSE admitted to each stroke center.

**Figure 1:**
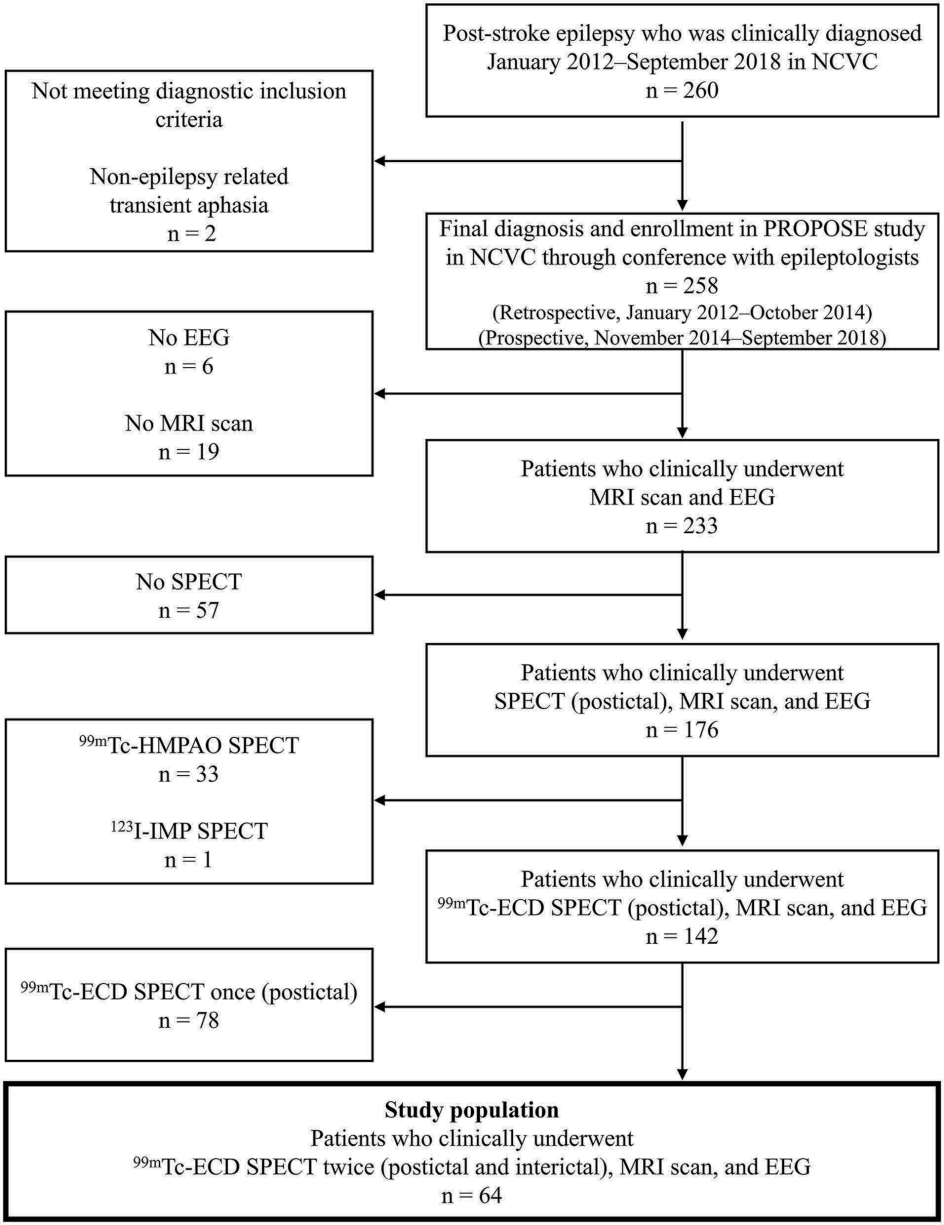
Patient enrollment. ECD, ethyl cysteinate dimer; EEG, electroencephalography; HMPAO, hexamethyl propylene amine oxime; IMP, iodoamphetamine; NCVC, National Cerebral and Cardiovascular Center.

The flow chart of the study protocol is presented in Figure 1. We retrospectively selected 260 consecutive patients admitted to the Department of Stroke and Cerebrovascular Diseases in the NCVC and clinically diagnosed with PSE between January 2012 and September 2018. A routine-length (~20 min) scalp EEG was interpreted by one or more epileptologists (MT, KK, and AS). The final diagnosis of PSE for enrollment in PROPOSE study was verified at the consensus conference attended by two or more neurologists and one or two epileptologists (MT, KK, and AS) with reference to seizure history, semiology, EEG findings, therapeutic response, and clinical course. All enrolled patients met the new ILAE clinical definition of epilepsy (12), having at least one unprovoked seizure more than 7 days after index stroke (i.e., late seizure). Two hundred and fifty-eight patients were diagnosed and enrolled in the study through the consensus conference in NCVC. Among them, 233 patients underwent routine-length scalp EEG and MRI scans. Finally, 64 patients having postictal and interictal ^99m^Tc-ethyl cysteinate dimer (ECD)-SPECT were enrolled as the study population.

The present study was approved by the ethics committee of the NCVC (ethical approval number M26-093-10). Written informed consent for research, including initial and follow-up SPECT, was provided by patients. If written informed consent was not available before SPECT imaging (for example, from disability or impaired consciousness), orally informed consent preceded written consent through an opt-out process. An opt-out choice was also displayed on the center website. No patients subsequently refused to participate in the study.

### Evaluation of Electroencephalogram and Seizure Semiology

The localization of epileptiform abnormalities in all scalp EEGs was evaluated by two board-certified epileptologists who were blind to clinical details. Epileptiform EEG findings were defined as interictal epileptiform (spike and sharp waves), periodic, or ictal epileptiform discharges based on the American Clinical Neurophysiology Society guidelines and consensus statements (13, 14), and the Salzburg EEG criteria (15, 16). The laterality of the symptomatogenic zone, the cortical region producing ictal symptoms, was determined in reference to semiology, lateralizing signs, such as unilateral tonic/clonic seizure, eye and head version, and aphasia (17). We localized all patterns to 5 pre-defined regions (frontal, central, parietal, temporal, and occipital) in each hemisphere. We considered the Fp1/2 and F3/4/z as the frontal, C3/4/z central, P3/4/z parietal, F7/8 and T3/4/5/6 temporal, and O1/2 occipital region.

### Evaluation of Magnetic Resonance Imaging

All patients in the study populations underwent magnetic resonance angiography (MRA) and MRI studies, including fluid-attenuated inversion recovery (FLAIR), diffusion-weighted imaging, and T2^*^-weighted image sequences, which were performed on admission (3T, Magnetom Verio or Spectra; Siemens Medical Solutions, Erlangen, Germany). All MR images were assessed independently by a neuronuclear medicine specialist (KF) and expert neuroradiologists (YM) blinded to all clinical information to determine stroke etiology and exclude acute stroke. FLAIR images were obtained and used for the co-registration of SPECT images. Sequence parameters of FLAIR images were as follows: FOV, 230 × 202 mm; acquisition matrix, 320 × 182 or 320 × 189; slice thickness, 5.0 mm; interslice gap, 1.0 mm; echo time, 94 to 114 ms; and repetition time, 12 000 ms.

### SPECT Data Acquisition

The protocols for SPECT data acquisition were as follows. Patients were placed in a supine position with their eyes closed in a quiet and dimly lit environment for 5 min and 16.2 mCi (600 MBq) of ^99m^Tc-ECD was injected intravenously. During the scan, no abnormal behaviors or subjective manifestations of seizures were reported. The imaging data were acquired using a dual-headed gamma camera (ECAM, Siemens) 15 min after injection of the tracer, with a scan duration of 20 min. The field of view of the image contained the entire brain, including the cerebellum. Projection data were processed with a filtered back projection, and the Chang attenuation correction was applied. SPECT axial images were reconstructed with a 176 × 176 matrix, 0.4 mm in-plane resolution, and a slice thickness of 0.48 mm.

### SPECT Data Evaluation

**Figure 2:**
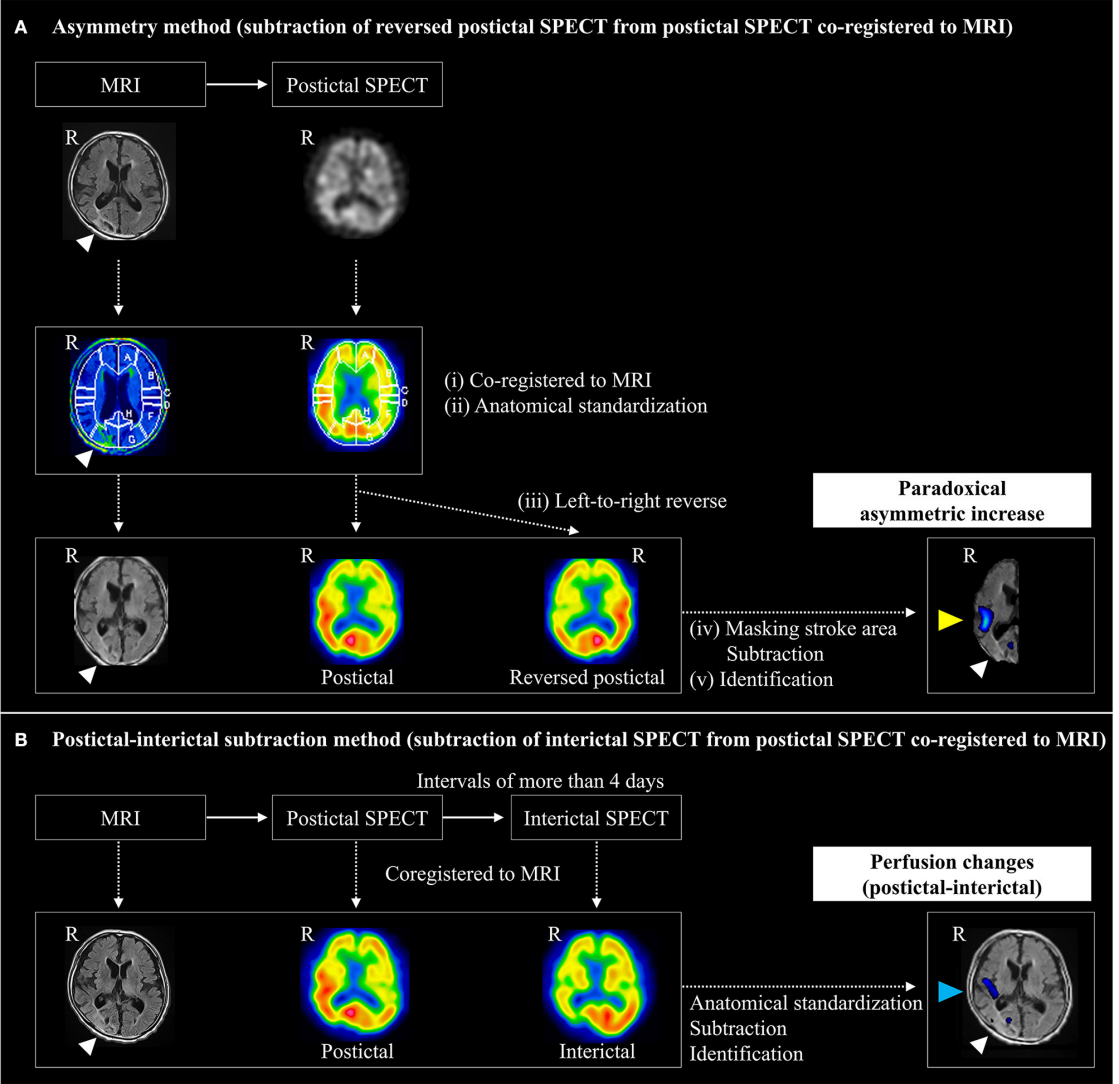
Imaging analysis protocol for detecting prolonged postictal hyperperfusion. Postictal hyperperfusion was evaluated by the following two methods: **(A)** the asymmetry method and **(B)** the postictal-subtraction method. **(A)** The asymmetry method was devised for detecting a higher perfusion area (yellow arrowhead) in the hemisphere ipsilateral and adjacent to stroke lesions (white arrowhead), compared to the corresponding contralateral area, which was defined as a paradoxical asymmetric increase by subtracting anatomically standardized non-reversed SPECT imager from left-to-right reversed postictal SPECT images to obtain non-reversed – reversed difference. **(B)** The postictal-interictal method was devised based on detecting an area of perfusion postictal-interictal changes (arrowed in blue): anatomical standardized postictal and interictal SPECT images were subtracted to obtain the postictal – interictal difference. R, right.

The imaging analysis protocol is shown in Figure 2. We evaluated postictal hyperperfusion using the following two methods: (a) asymmetry and (b) P-I subtraction. Perfusion changes were independently assessed by a neuronuclear medicine specialist (KF) and a stroke neurologist (TK), blinded to clinical and EEG information. Interobserver inconsistencies were resolved through consensus meetings. Each observer (KF and TK) performed the assessment two times, with an interval to measure intraobserver reliability.

(a) Asymmetry method using only postictal SPECT images (subtraction analysis of reversed postictal SPECT from postictal SPECT co-registered to MRI) (Figure 2A).
  We formulated a novel asymmetry method for detecting postictal hyperperfusion using only postictal SPECT images. This method was devised by detecting a higher perfusion area in the hemisphere ipsilateral and adjacent to stroke lesions, compared to the corresponding contralateral area, defined as paradoxical asymmetric increase by the following process.
  (i) FLAIR images were converted from 5 to 2 mm slice-thick images and postictal SPECT images from each patient reconstructed and co-registered to each FLAIR image, using an automated image registration (AIR) program (18).
  (ii) MRI was anatomically standardized on a three-dimensional stereotaxic template (3DSRT) from each patient using the FLAIR template (19), and the parameters extracted. Using the same parameter, postictal SPECT scans were also anatomically standardized from each patient.
  (iii) The left-to-right reversed images were obtained to inverse the postictal images at the midline using a Daemon Research Image Processor (DRIP; Fujifilm Toyama Chemical Co., Ltd., Tokyo, Japan).
  (iv) Subtraction analysis of reversed postictal, from postictal, SPECT, co-registered to MRI (20), was performed using automatic software (Fujifilm Toyama Chemical Co., Ltd., Tokyo, Japan) by the following process. SPECT images were smoothed with a 10-mm full width, at half of the maximum Gaussian filter. The stroke areas were removed from MRI by applying a binary mask, created from the non-reversed postictal SPECT images partly automatically, with the threshold set to 30% of the maximum value of the whole brain, using DRIP software without manual definition. The outer scalp was removed from MRI by applying another binary mask for the whole brain, except stroke areas (21). Each voxel count in the non-reversed and reversed postictal SPECT images was normalized according to global mean voxel counts, except in stroke areas. Anatomical standardized non-reversed and reversed postictal SPECT images were subtracted to obtain non-reversed–reversed difference. The mean and standard deviations of global change in perfusion were calculated in the subtraction image of each patient, except in stroke areas. The cluster extent threshold was set to 125 and voxels with counts above one and a half and two standard deviations were measured. Brain regions with a significant increase in perfusion were superimposed on tomographic MRI images.
  (v) Finally, we identified paradoxical asymmetric increase, defined as the higher perfusion area adjacent to stroke lesions, in the subtraction images with a threshold above two standard deviations. We excluded artifacts lying outside the cortical surface and inside ventricles, and in brain regions distant at intervals of more than 5 cm, or contralateral from the stroke lesions. Those without high perfusion area adjacent to stroke lesions in the images, with a threshold of two standard deviations, had an identification above one and a half standard deviation added.
(b) Postictal–interictal subtraction method using postictal and interictal SPECT images (subtraction analysis of interictal from postictal SPECT co-registered to MRI) (Figure 2B).

We identified perfusion changes on postictal, referenced to interictal, SPECT using a modified method from subtraction ictal SPECT co-registered to MRI (SISCOM) method (20), as described previously (4). Briefly, normalized postictal and interictal SPECT images were subtracted to obtain the postictal-interictal difference. Then, brain regions with a significant increase and decrease above two standard deviations in perfusion were superimposed on tomographic images of MRI. Finally, we identified postictal-interictal perfusion changes.

### Localization of Perfusion Changes on Postictal SPECT and Structural Lesions on MRI

We visually localized perfusion changes on postictal SPECT and structural lesions (i.e., stroke lesions) on MRI spatially normalized onto the segmented template. To evaluate consistency between imaging and EEG data, brain regions were classified into five areas in each hemisphere: frontal (superior, middle, inferior, and orbital gyrus), central (precentral and postcentral gyrus), parietal (superior and inferior parietal lobules, and precuneus), occipital (cuneus, lingual gyrus and superior, middle, and inferior occipital gyrus), and temporal (superior, middle, and inferior temporal gyrus, Heschl's gyrus and temporal pole) areas. If two or more regions were involved, localization was classified into multiple areas.

### Statistical Analysis

We calculated the detection rates of postictal hyperperfusion on the asymmetry and P-I subtraction methods. Time intervals from seizure end-to-initial SPECT and EEG were calculated. The Wilcoxon rank-sum test was performed to analyze the relationship between the time intervals and incidence of perfusion changes using the asymmetry method. Calculations of sensitivity and specificity were conducted, and a receiver operating characteristic (ROC) curve was plotted to evaluate and compare the diagnostic capabilities of both methods. Sensitivity, specificity, ROC curves, and areas under the curve were calculated with the assumption that the P-I subtraction method is the gold standard for the determination of hyperperfusion. ROC curves were plotted using a categorical variable with only two possible outcomes (positive and negative), which may have led to underestimation of the area under the curve.

The laterality of the maximum perfusion changes of the asymmetry method was compared with that of the symptomatogenic zone and epileptiform EEG findings. Furthermore, the localization of the maximum perfusion changes of the asymmetry method was also compared with that of the epileptiform EEG findings.

Previous reports in temporal lobe epilepsy showed variable perfusion changes could occur during the postictal phase, except for PSE (22, 23), including hypoperfusion contralateral to the focus after secondary generalization [currently described as “focal to bilateral tonic-clonic seizures” (FBTCS)] (22). Thus, if postictal hypoperfusion occurs in the hemisphere contralateral to the stroke lesion in PSE, the phenomenon may lead to pseudo-positive findings on the asymmetry method. Hence, we investigated whether patients with positive findings with the asymmetry method had contralateral postictal hypoperfusion with the P-I subtraction method. We also assessed the association between FBTCS and the asymmetry method data. Statistical analysis was performed using JMP14 software (SAS Institute, Cary, NC, USA).

### Data Availability

The scripts used for data preprocessing and anonymized data are available upon reasonable request.

## Results

### Patient Characteristics

Patient characteristics are shown in Table 1. In the study population of the 64 patients, the median age was 75 years (range, 38–95 years), and 39 (61%) were male. The study population included patients with cerebral infarction (*n* = 31), intracerebral hemorrhage (*n* = 29), and subarachnoid hemorrhage (*n* = 4); three patients had multiple stroke subtypes. Sixty of the sixty-four patients (94%) presented lateralizing, and 32 (50%) epileptiform EEG signs. The median time interval from seizure end to postictal SPECT was 21.0 h (range, 2.2–120 h). The time intervals from seizure end to initial EEG were significantly shorter than those from seizure end to initial SPECT (median, 18.9 vs. 21.0 h, *p* < 0.001, Wilcoxon signed-rank test).

**Table 1:** Baseline characteristics.

**Baseline characteristics**	**Total (*n* = 64)**
Age in years	75 (38–94)
Male, *n* (%)	39 (61)
Time elapsing from stroke onset to late seizure in years	1.08 (0.04–22.3)
Newly diagnosed epilepsy, *n* (%)	41 (64)
Recurrent epilepsy, *n* (%)	23 (36)
Pre-administration AED^a^	19/23
Frequency of seizures per year	1 (0.2–2)
Status epilepticus, *n* (%)	21 (33)
Seizure symptoms	
Hemi-convulsion, *n* (%)	28 (44)
Paresis, *n* (%)	16 (25)
Aphasia, *n* (%)	11 (17)
Version of eyes/head, *n* (%)	37 (58)
Lateralizing sign, *n* (%)	55 (86)
Non-convulsive seizure, *n* (%)	22 (34)
Focal seizure	64 (100)
Focal-onset impaired awareness seizure, *n* (%)	60 (94)
Focal to bilateral tonic-clonic seizure, *n* (%)	22 (34)
**MRI findings**	
Stroke subtypes	
i) Cerebral infarction, *n* (%)	31 (48)
Hemorrhagic infarction	14/31
ii) Intracerebral hemorrhage, *n* (%)	29 (45)
Lobar	17/29
Putamen	11/29
Thalamus	1/29
iii) Subarachnoid hemorrhage, *n* (%)	4 (6.3)
**Electroencephalography findings**	
Interictal/ictal epileptiform discharges or periodic discharges, *n* (%)^b^	32 (50)
Interictal epileptiform discharges, *n* (%)	24 (38)
Periodic discharges, *n* (%)	18 (28)
Ictal epileptiform discharges *n* (%)	4 (6.3)
Number of EEG studies	2 (1–8)
Time interval (seizure end–initial EEG), h	18.9 (0–92.5)
**Timing of SPECT scans**	
Time interval (seizure end–initial SPECT), h	21 (2.2–120)
Time interval (initial SPECT–second SPECT), h	6.9 (3.9–21)

*Data are presented as n (%) or median (range)*.

*^a^Patients who received antiepileptic drugs before admission yet to be diagnosed*.

*^b^Twelve patients had two or three types of epileptiform abnormalities*.

*AED, antiepileptic drug EEG, electroencephalography; h, hour; ICH, intracerebral hemorrhage; SAH, subarachnoid hemorrhage*.

### Diagnostic Accuracy and Lateralization Concordance With Other Clinical Findings of the Asymmetry Method

**Figure 3:**
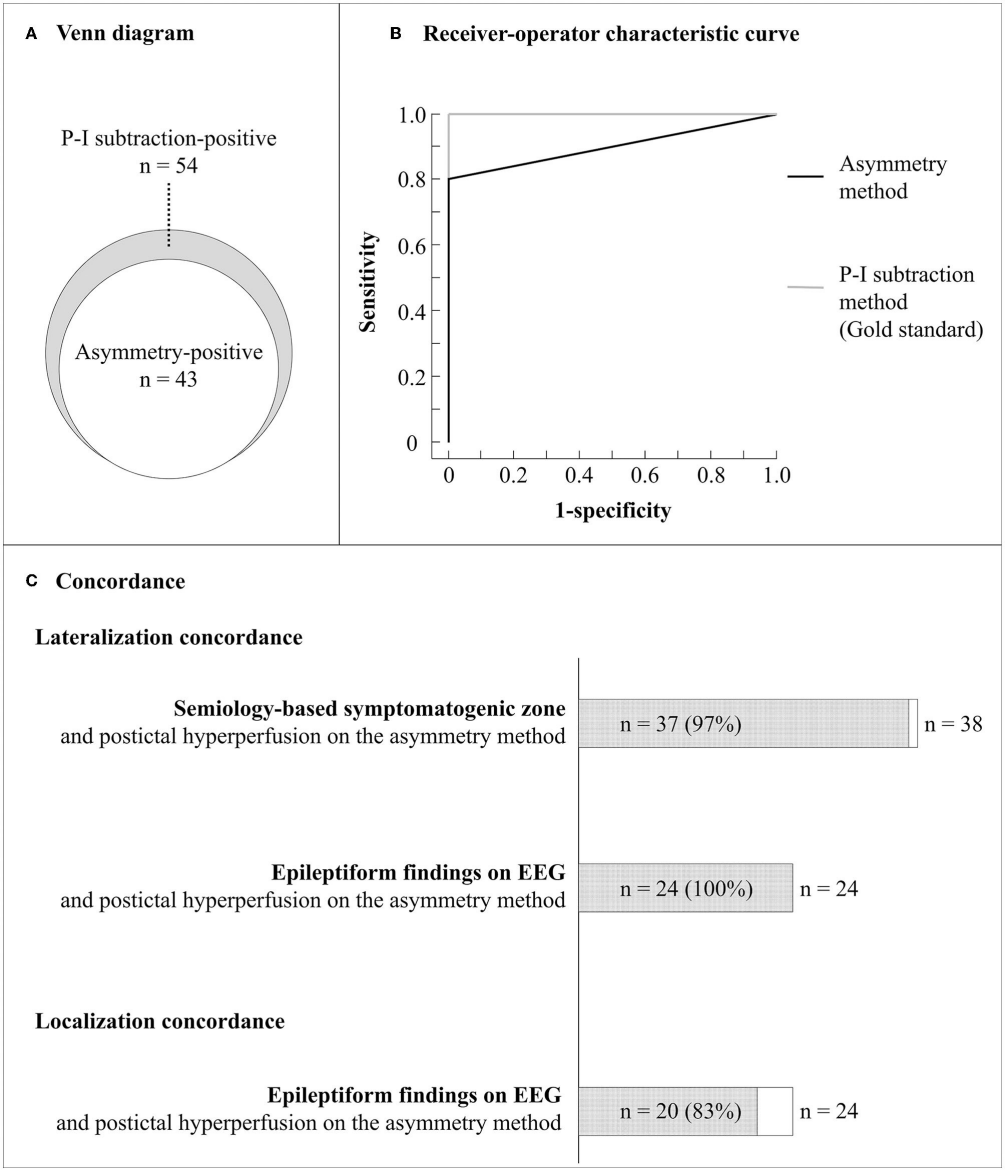
Diagnostic accuracy and lateralization concordance with other clinical findings of the asymmetry method. **(A)** Venn diagram for the relationships between the patients with positive results on the asymmetry and P-I subtraction methods. **(B)** Receiver operating characteristic curves for the asymmetry and P-I subtraction methods. **(C)** Lateralization and localization concordance between clinical findings. Semiology-based symptomatogenic zone was identified in the same hemisphere in 37 of 38 patients with lateralizing signs (97%). Epileptiform findings were detected on EEG in 24 of the 43 patients and observed in the same hemisphere in 24 of the 24 patients (100%) and in the concordant regions in 20 of the 24 patients (83%). EEG, electroencephalography; P-I, postictal-interictal.

Among the enrolled 64 patients, 43 (67%) had postictal hyperperfusion using the asymmetry method, 35 had increased perfusion above two, and the remaining 8 above one and a half standard deviations. Among the 64 patients, 54 (84%) had postictal hyperperfusion on the P-I subtraction method. There was no difference in the time intervals from seizure end to initial SPECT in patients with and without hyperperfusion using the asymmetry method [median, 20.5 h (range, 2.2–120 h) vs. median, 22.5 h (range, 3.9–111.4 h), *P* = 0.56]. Figure 3A shows a Venn diagram for the relationships between the patients with positive results on the two methods. All patients with positive results using the asymmetry method had positive results with the P-I subtraction method.

The intraobserver reliability of the asymmetry method for hyperperfusion was 98.4%, with a Kappa statistic of 094; while interobserver agreement for the region with hyperperfusion was 96.9%, with a Kappa statistic of 0.88. The intraobserver reliability of the P-I subtraction method for hyperperfusion was 98.4%, with a Kappa statistic of 0.94; while interobserver agreement for the region with hyperperfusion was 96.9%, with a Kappa statistic of 0.88. Taking the P-I subtraction method as gold standard, the diagnostic accuracy of the asymmetry method showed sensitivity and specificity between 80 and 100%. Figure 3B shows ROC curves for the asymmetry and P-I subtraction methods. The area under the ROC curve for the asymmetry method was 0.91. The laterality of the maximum hyperperfusion area on the asymmetry method had a high concordance rate with symptomatogenic zone (97%) and epileptiform EEG findings (100%). Further, the brain area with maximum hyperperfusion on the asymmetry method corresponded to that of epileptiform findings on EEG in 20 of 24 patients who had both hyperperfusion and epileptiform EEG findings (83%) (Figure 3C).

### Perfusion Patterns and Secondary Generalization

Among the 43 patients with positive findings in the asymmetry method, P-I subtraction showed hyperperfusion in the side ipsilateral to stroke lesion in all patients. In contrast, the P-I subtraction method showed no hypoperfusion in the areas located on the opposite side (across the median) of the positive areas with the asymmetry method. Furthermore, there was no significant difference in the detection rate of postictal hyperperfusion with the asymmetry method between the patients with and without FBTCS (55 vs. 74%, *p* = 0.12).

## Discussion

This study established a novel objective asymmetry method for the detection of prolonged hyperperfusion using a single postictal SPECT in PSE. The asymmetry method had a comparable diagnostic accuracy with 80% sensitivity and 100% specificity, considering the P-I subtraction method as a gold standard.

The present study showed the asymmetry method could detect hyperperfusion on postictal SPECT, based on the premise of “paradoxical asymmetric increase.” Previous studies have shown cerebral blood flow level in areas ipsilateral and adjacent to stroke lesions decreases below that in corresponding contralateral areas, namely, an asymmetric decrease, during the chronic stroke phase, including in areas without morphological abnormality on MRI (10, 11). The present study showed the asymmetry method could detect paradoxical asymmetric increases in areas ipsilateral and adjacent to stroke lesions beyond the corresponding contralateral area on postictal SPECT scans.

Objectivity is an issue for asymmetry analysis. Our protocol includes a novel objective technique, namely subtraction analysis of reversed postictal, from postictal SPECT co-registered to MRI. This procedure requires no manual definition unlike an asymmetry index technique, which requires region of interest definition (6–9). Consequently, the present study yielded high intra- and inter-observer agreements (Kappa statistic of 0.94 and 0.88, respectively) to a similar extent as the P-I subtraction method, ensuring high objectivity and reproducibility.

The asymmetry method had a comparable diagnostic accuracy with 80% sensitivity and 100% specificity to the gold standard P-I subtraction method. In addition, using the asymmetry method, the prolonged hyperperfusion areas had high laterality correspondence with ictal semiology (97%) and epileptiform EEG findings (100%). As described in our recent paper (4), this high concordance implies prolonged hyperperfusion, representing epileptic propagation in the hemisphere ipsilateral to stroke lesions. Moreover, the asymmetry method also permits a wide diagnostic time window in PSE of hours or even days after seizure end. The advantages of the asymmetry method also include reduced radiation exposure and cost compared with the P-I subtraction method, which requires both postictal and interictal SPECT scans. The asymmetry method thus possesses clinical and financial advantages, coupled with accurate detectability for postictal hyperperfusion.

Relatively high sensitivity was found, but hyperperfusion was not detected in 12 patients (20%) using the asymmetry but not the P-I method. This suggests possible factors preventing perfusion increase in the ipsilateral side of stroke lesions during the postictal phase. The influence of clinical factors on perfusion suppression should be examined in future studies to improve the sensitivity of the asymmetry method.

This study has limitations. We assessed patients who underwent SPECT two times clinically only. This may have introduced selection bias as SPECT scans tend to be repeated for patients with epilepsy difficult to diagnose, such as those with non-convulsive status epilepticus. However, the detection of postictal hyperperfusion using SPECT may be useful in such patients. Moreover, we used SPECT to measure cerebral perfusion, a modality not always available in routine clinical practice. However, our novel method may be applicable to other imaging modalities, including advanced arterial spin labeling MRI and computer tomography perfusion imaging.

In summary, the present study developed a novel objective asymmetry method for the detection of prolonged hyperperfusion using postictal SPECT scan in PSE. Asymmetry is comparable to the P-I method but possesses significant advantages with only a single scan required, along with reduced radiation exposure and cost.

## Data Availability Statement

The raw data supporting the conclusions of this article will be made available by the authors, without undue reservation.

## Ethics Statement

The studies involving human participants were reviewed and approved by the Ethics Committee of the NCVC (approval number M26-093-10). The patients/participants provided their written informed consent to participate in this study.

## Author Contributions

KF: study conception and design, data acquisition, analysis and interpretation, and drafting of the paper. TT: study design and imaging data analysis and interpretation. ST: study design and critical revision of the manuscript. MT, KK, and AS: EEG analysis and interpretation and diagnosis. YM: imaging data analysis and interpretation. KN: statistical analysis. MK, KT, RM, and RT: critical revision of the manuscript. AI: data interpretation and critical revision of the manuscript. MI: study conception and design, data interpretation, and critical revision of the manuscript. All authors approved the final version of the manuscript.

## Funding

The author(s) disclose receipt of the following financial support for the research, authorship, and/or publication of this article. This research was supported by the Practical Research Project for Lifestyle-related Diseases including Cardiovascular Diseases and Diabetes Mellitus from the Japan Agency for Medical Research and Development (AMED#18ek0210057h0003). The study was also partially supported by the Japan Society for the Promotion of Science (JSPS) (19K16888 and 22K15721) and Taiju Life Social Welfare Foundation. Role of the Funder/Sponsor: AMED, JSPS, and Taiju Life Social Welfare Foundation had no role in the design and conduct of the study, collection, management, analysis, and interpretation of the data, preparation, review, or approval of the manuscript, or decision to submit the manuscript for publication.

## Conflict of Interest

AI belongs to the Department of Epilepsy, Movement Disorders and Physiology, an Industry-Academia Collaboration Course, supported by a grant from Eisai Corporation, Nihon Kohden Corporation, Otsuka Pharmaceutical Co., and UCB Japan Co. The remaining authors declare that the research was conducted in the absence of any commercial or financial relationships that could be construed as a potential conflict of interest.

## Publisher's Note

All claims expressed in this article are solely those of the authors and do not necessarily represent those of their affiliated organizations, or those of the publisher, the editors and the reviewers. Any product that may be evaluated in this article, or claim that may be made by its manufacturer, is not guaranteed or endorsed by the publisher.
